# Low vitamin D levels do not aggravate COVID-19 risk or death, and vitamin D supplementation does not improve outcomes in hospitalized patients with COVID-19: a meta-analysis and GRADE assessment of cohort studies and RCTs

**DOI:** 10.1186/s12937-021-00744-y

**Published:** 2021-10-31

**Authors:** Jie Chen, Kaibo Mei, Lixia Xie, Ping Yuan, Jianyong Ma, Peng Yu, Wengen Zhu, Chunhua Zheng, Xiao Liu

**Affiliations:** 1 Department of Cardiology, the First Hospital of Nanchang, Nanchang, 330006 Jiangxi China; 2 Department of Anaesthesia, the People’s Hospital of Shangrao, Shangrao, Jiangxi China; 3 Department of Respiratory and Critical Care Medicine, the Second Affiliated Hospital of Nanchang University, Nanchang, Jiangxi China; 4 Department of Cardiology, the Second Affiliated Hospital of Nanchang University, Nanchang, Jiangxi China; 5 Department of Pharmacology and Systems Physiology, University of Cincinnati College of Medicine, Cincinnati, USA; 6 Endocrine Department, the Second Affiliated Hospital of Nanchang University, Nanchang, Jiangxi China; 7 Department of Cardiology, The First Affiliated Hospital of Sun Yat-Sen University, Guangzhou, Guangdong China; 8 Department of Cardiology, The Sun Yat-Sen Memorial Hospital of Sun Yat-Sen University, Guangzhou, Guangdong China; 9 Guangdong Province Key Laboratory of Arrhythmia and Electrophysiology, Guangzhou, Guangdong China; 10 Guangzhou Key Laboratory of Molecular Mechanism and Translation in Major Cardiovascular Disease, Guangzhou, Guangdong China

**Keywords:** Vitamin D, COVID-19, Meta-analysis, Nutrition

## Abstract

**Background:**

The associations between vitamin D and coronavirus disease 2019 (COVID-19) infection and clinical outcomes are controversial. The efficacy of vitamin D supplementation in COVID-19 is also not clear.

**Methods:**

We identified relevant cohort studies that assessed the relationship between vitamin D, COVID-19 infection and associated death and randomized controlled trials (RCTs) that reported vitamin D supplementation on the outcomes in patients with COVID-19 by searching the PubMed, EMBASE, and medRxiv databases up to June 5th, 2021. Evidence quality levels and recommendations were assessed using the GRADE system.

**Results:**

Eleven cohort studies with 536,105 patients and two RCTs were identified. Vitamin D deficiency (< 20 ng/ml) or insufficiency (< 30 ng/ml) was not associated with an significant increased risk of COVID-19 infection (OR for < 20 ng/ml: 1.61, 95% CI: 0.92–2.80, I2 = 92%) or in-hospital death (OR for < 20 ng/ml: 2.18, 95% CI: 0.91–5.26, I2 = 72%; OR for < 30 ng/ml: 3.07, 95% CI: 0.64–14.78, I2 = 66%). Each 10 ng/ml increase in serum vitamin D was not associated with a significant decreased risk of COVID-19 infection (OR: 0.92, 95% CI: 0.79–1.08, I2 = 98%) or death (OR: 0.65, 95% CI: 0.40–1.06, I2 = 79%). The overall quality of evidence (GRADE) for COVID-19 infection and associated death was very low. Vitamin D supplements did not significantly decrease death (OR: 0.57, I2 = 64%) or ICU admission (OR: 0.14, I2 = 90%) in patients with COVID-19. The level of evidence as qualified using GRADE was low.

**Conclusions:**

Current evidence suggested that vitamin D deficiency or insufficiency was not significantly linked to susceptibility to COVID-19 infection or its associated death. Vitamin D supplements did not significantly improve clinical outcomes in patients with COVID-19. The overall GRADE evidence quality was low, we suggest that vitamin D supplementation was not recommended for patients with COVID-19.

**Supplementary Information:**

The online version contains supplementary material available at 10.1186/s12937-021-00744-y.

## Introduction

Recent studies have highlighted that the mean plasma vitamin D level is significantly lower in patients who tested positive for COVID-19 than in patients who tested negative [1]. Rhodes et al. [2] found that patients with COVID-19 residing in all countries that lie below 35 degrees north latitude have relatively low death. These results suggest that low vitamin D levels are associated with increased COVID-19 infection rates and worse outcomes. Several studies have reported that serum vitamin D deficiency is associated with an increased risk of COVID-19 positivity and worse outcomes (e.g., severe COVID-19 and in-hospital death) [3–5]. However, a larger cohort-based study from the UK Biobank showed a nonsignificant association after adjustment for confounders (COVID-19 infection HR: 1.00; *p* = 0.89; death HR: 0.98; *p* = 0.69) [6]. Two other studies also found no association between vitamin D and COVID-19 positivity [7, 8]. Therefore, the impact of vitamin D on COVID-19 risk and clinical outcomes remains controversial, and a definite conclusion has not been reached. Thus, we performed a meta-analysis to clarify the association between serum vitamin D level, COVID-19 risk and associated death and assessed the effect of vitamin D supplements on clinical outcomes in patients with COVID-19 by pooling current evidence from clinical trials.

## Methods

The present study was performed according to Preferred Reporting Items for Systematic reviews and Meta-Analyses Statement (PRISMA) guidelines (Supplemental Table S1). The protocol of this meta-analysis was not registered.

### Literature search and study selection

Two authors (X.L. and J.C.) independently searched several databases (PubMed, EMBASE, and medRxiv) using the following groups of keywords with no language restrictions up to June 5th, 2021,: 2019-novel coronavirus, SARS-CoV-2, COVID-19, 2019-nCoV, vitamin D, death, severe and ICU. The details of the search strategy are described in Supplemental Table S2. We also searched the reference lists of relevant publications to identify further studies. The following inclusion criteria were used: 1) human studies that were published as original articles; 2) reports designed as cohorts with estimated effects (multivariate-adjusted) and 95% confidence interval (CI) results that reported the association between vitamin D, COVID-19 risk, and death; and 3) clinical trials (randomized controlled design) that assessed vitamin D supplementation on clinical outcomes in patients with COVID-19. Case-control, cross-sectional were excluded. When multiple papers reporting on the same study were identified, the most informative or complete article was included.

### Data extraction and statistical analysis

Two authors independently extracted all data from the included studies. Discrepancies and disagreements were resolved via collegial discussion. The following information was extracted: first author, country, publication year, gender, mean or median age, study design, sample size, vitamin D level, OR or RR with the 95% CI for each category (results adjusted according to most potential confounders), and adjusted variables.

Effect measures were transformed to their natural logarithms (logOR), and the standard errors (SElog[OR]) were calculated from the corresponding 95% CI. We calculated study-specific slopes (vitamin D per 10 ng/ml increase), and 95% CIs from the natural logs of the reported RRs and CIs across categories of vitamin D levels [9, 10]. Cochran Q and I2 statistics were used to detect statistical heterogeneity between studies. The overall quality of the included studies was assessed with the Newcastle-Ottawa quality assessment scale (NOS), and an NOS score ≥ 6 was considered high quality [11]. The risk of bias for the trials was assessed with the Cochrane risk of bias tool. The GRADE methodology was used to evaluate the quality of the body of retrieved evidence (GRADEpro, https://gdt.gradepro.org/app/#projects). All statistical analyses were performed using Review Manager version 5.3 (The Cochrane Collaboration 2014; Nordic Cochrane Center Copenhagen, Denmark). All statistical tests were double-sided, and *P* < 0.05 was considered statistically significant.

## Results

### Study selection

**Fig. 1:**
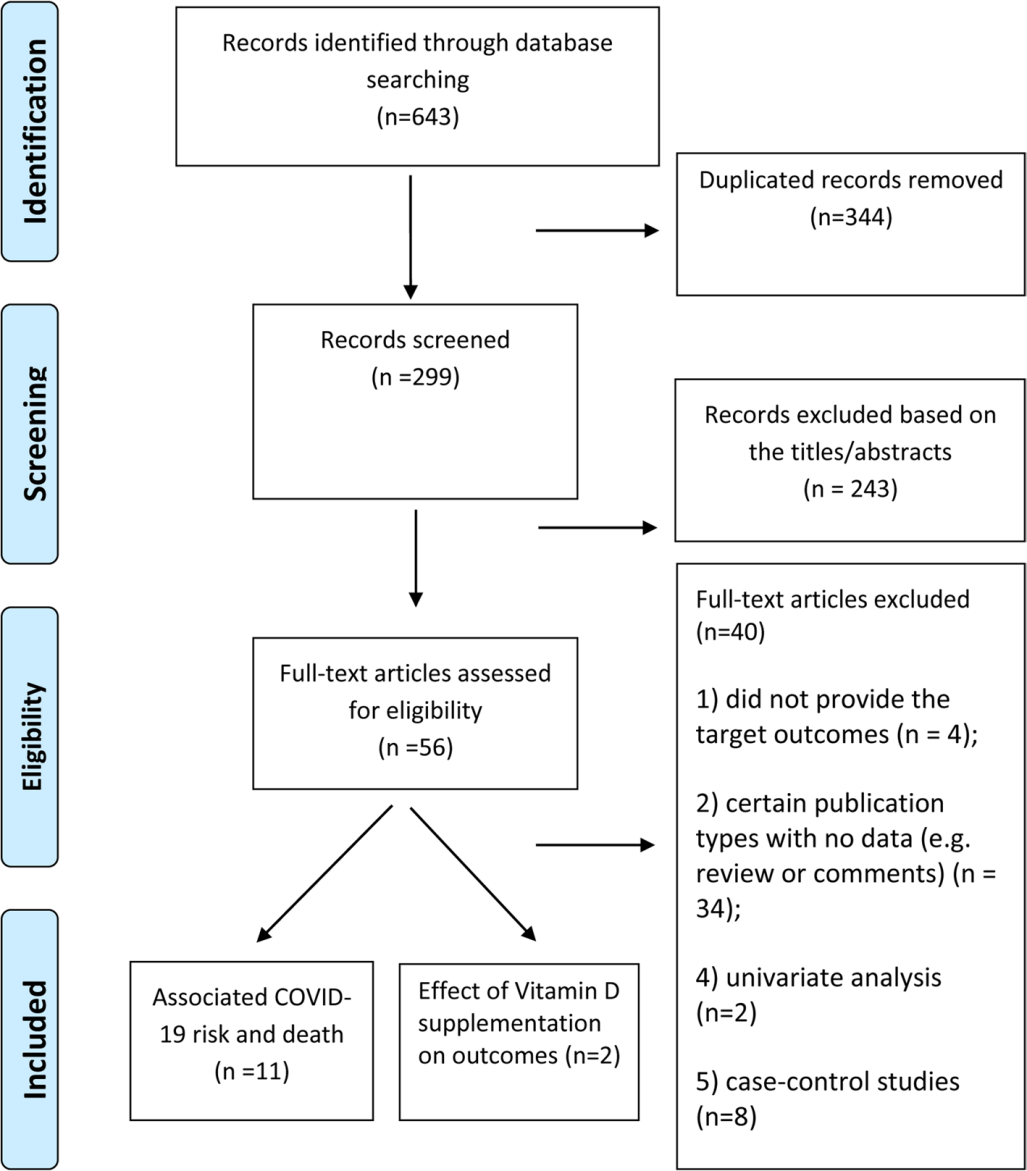
Flow chart of study selection in this meta-analysis

As shown in Fig. 1, we identified 643 studies in the initial database search. After the removal of duplicates (*n* = 344) and studies with insufficient information (*n*=283) on vitamin D and COVID-19, a total of 13 [3, 5, 6, 12–21] studies were included.

**Fig. 2:**
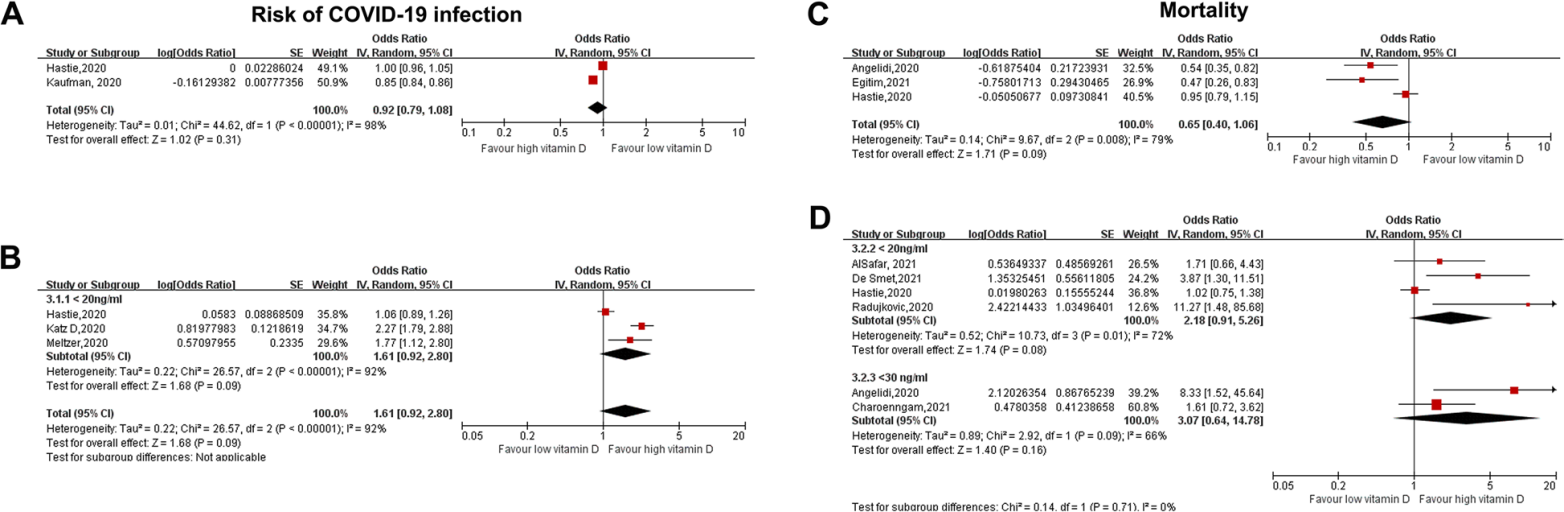
Forest plot showing the association between serum vitamin D level and risk of COVID-19 infection and death in patients with COVID-19. **A-B:** COVID-19 infection, vitamin D was analyzed as a categorical variable (**A**: upper) or continuous variable (**B**: lower). **C-D**: Death, vitamin D was analyzed as a categorical variable (**C**: upper) or continuous variable (**D**: lower). (Continuous variable: vitamin D per 10 ng/ml increase). Abbreviations: COVID-19, coronavirus disease 2019; OR, odds ratio; CI, confidence interval; IV, inverse variance; SE, standard error

### Study characteristics and quality

Each study is listed in Table 1. Of the 13 studies, 11 [3, 5, 6, 12–15, 17–19, 22] cohorts with 536,105 patients assessed the COVID-19 risk and death, and two [16, 20] RCTs investigated the effect of vitamin D supplementation on outcomes in patients with COVID-19. The mean patient age ranged from 49 to 69 years. Ten [3, 5, 12–15, 17–19, 22] studies were based on hospitalized patients, and one [6] study was based on a prospective cohort (UK Biobank). Most studies [3, 14, 18–20, 22] (*n* = 6) were performed in the US, 5 [5, 6, 13, 15, 16] studies were conducted in Europe, 1 [17] study was performed in Asia, and 1 [12] study was performed in Australia. Four [3, 6, 23, 24] studies reported vitamin D levels and COVID-19 positivity, and eight [5, 6, 12–15, 17, 22] studies reported the association between vitamin D levels and death. The overall quality of the observational studies was acceptable (NOS score ≥ 6) (Supplemental Table S4). Among the two RCTs were [16, 20] had a low risk of bias (Supplemental Table S5).

**Table 1:** Basic characteristics of included articles reporting the association between vitamin D and COVID-19 infection and death, effect of vitamin D supplement on clinical outcomes in patients with COVID-19

**Author, publication year, country**	**Study design, Follow up**	**Source of patients**	**Sample size (N)**	**Mean age (years), male**	**Definition of Vitamin D exposure**	**Expose level of vitamin D**	**OR (95% CI)**^**a**^	**Adjustments for confounders**
Hastie, 2020, UK [6, 8]	Prospective cohort study	UK Biobank	341,484	49, 48%	Conducted between 2006 and 2010	Per 10 ng/ml < 20 ng/ml > 20 ng/ml Per 10 nmol/L < 20 ng/ml > 20 ng/ml	COVID-19 infection 1.00 (0.89–1.12) 1.06 (0.89–1.26) Ref Death 0.95 (0.79–1.15) 1.02 (0.75–1.38) Ref	Age, sex, ethnicity, month of assessment, Townsend deprivation quintile, household income, BMI category, smoking status, diabetes, systolic blood pressure, diastolic blood pressure, self-reported health rating, and long-standing illness, disability or infirmity
Hastie, 2020, UK [6, 8]	Retrospective cohort study	University of Chicago Medicine	498	49,25%	Within 1 year before their first COVID-19 tests	< 20 ng/ml > 20 ng/ml	COVID-19 infection 1.77 (1.12–2.81) Ref	Age, sex, ethnicity, race, employee status. Hypertension, DM, chronic pulmonary disease, pulmonary circulation disorders, depression, CKD, liver disease, comorbidities with immunosuppression, BMI
Radujkovic,2020, Germany [5]	Retrospective cohort study	Medical university Hospital of Heidelberg	185	60,51%	At the time of admission and SARS-CoV-2 testing	< 20 ng/ml > 20 ng/ml	Death 11.27 (1.48–85.55) Ref	Age, gender, and any comorbidities
Charoenngam,2021, US [14]	Retrospective cohort study	Boston University Medical Center	287	62,47%	Measured at within 48 h after admission	< 30 ng/ml > 30 ng/ml	Death Ref 0.62 (0.28–1.41)	Age, sex, BMI, insurance, race, smoking, alcohol drinking, type 2 DM, hypertension, dyslipidemia, CAD, cerebrovascular disease, COPD, asthma, CKD, ESRD, malignancy, HIV infection, and heart failure.
Carpagnano, 2020, Italy [13]	Retrospective cohort study	Hospital Policlinic of Bari	42	NA	At admission	< 10 ng/ml > 10 ng/ml	Death 5.68 (1.14–28.97) Ref	Age, higher levels of creatinine, troponin, and IL-6
Katz D,2020, US [18]	Retrospective cohort study	UF health centers	887	NA, NA	October 1, 2015, through June, 30, 2020, for vitamin D deficiency	< 20 ng/ml > 20 ng/ml	COVID-19 infection 2.27 (1.79–2.87) Ref	Age, sex, malabsorption, PA, dental diseases, race, periodontal disease status, DM, obesity
Kaufman, 2020, US [19]	Retrospective cohort study	National clinical laboratory	191,779	54, 22%	Most recent vitamin D level	Per 10 ng/ml	COVID-19 infection 0.85 (0.84–0.86)	Male, northern and central latitudes, predominately black non-Hispanic zip codes, and predominately Hispanic zip codes
Angelidi,2020, US [22]	Retrospective cohort study	2 tertiary academic medical centers	144	66, 64%	Hospital personnel at Regular intervals	< 30 ng/ml > 30 ng/ml Per 10 ng/ml	Death 8.33 (1.6–50) Ref 0.54(0.35–0.82)	Age, BMI, ARB or ACEI, in-hospital drug treatment, CRP, smoking, heart failure, CAD, diabetes, hypertension, C-reactive protein level, and corticosteroids
De Smet,2021, Germany [15]	Retrospective cohort study	AZ Delta General Hospital	186	69,58%	Measured in patients with COVID-19 on admission and within 24 h	< 20 ng/ml > 20 ng/ml	Death 3.87(1.30–11.55) Ref	Age, higher CT severity score, presence of chronic lung
AlSafar, 2021, Australia [12]	Retrospective cohort study	Abu Dhabi, or Rashed hospital in Dubai.	464	46.6,80%	At recruitment	< 20 ng/ml > 20 ng/ml	Death 1.71 (0.66, 4.43) Ref	Age, sex, and comorbidities, BMI
Karahan, 2021, Turkey [17]	Retrospective cohort study	Health Sciences University	149	64, 54%	NA	Per 10 ng/ml	Death 0.93(0.88–0.98)	Age, smoking, hyperlipidemia, DM, CKD, Chronic AF, congestive heart failure, acute kidney injury, CRP, lymphocyte count, white blood cell count, serum albumin
**Author, publication year, country**	**Design**	**Sample size (male%); mean age(years)**	**Number of participants in intervention and control groups**	**25(OH)D assay**	**Mean baseline 25(OH)D concentrations, nmol/L (SD)**	**Oral dose of vitamin D in the intervention group**	**Control group**	**Outcome**
Murai,2021, US [20]	Multicenter double-blind RCT	240(56),56.3	intervention = 120; control = 120	NA	21.0 (10.2) 20.6 (8.1)	Single dose of 200,000 IU	Placebo	Death Admission to ICU
Castillo,2020, Spain [16]	Signal center RCT	76(59%),53.0	intervention = 50; control = 26	NA	NA	Single dose of oral calcifediol (0.532 mg)	Without Calcifediol treatment	Death Admission to ICU

*OR* odd ratio, *UCLA* University of California Los Angeles, *UK* United Kingdom, *US* Unite Status, *SES* residential socioeconomic status, *CKD* chronic kidney diseases, *ICU* intensive care unit, *AF* atrial fibrillation, *BMI* body mass index, *ARB* Angiotensin Receptor Blocker, *ACEI* angiotensin converting enzyme inhibitors, *CRP* C-reactive protein, *DM* diabetes mellitus, *CAD* coronary heart disease, *NA* not available, *RCT* randomized controlled trial, *ESRD* end-stage renal disease, *HIV* human immunodeficiency virus

^a^Hazard ratio and incidence rate ratio were treated as odd ratio

### The effect of low vitamin D level on COVID-19

The categorical analysis revealed that vitamin D deficiency (< 20 ng/ml) or insufficiency (< 30 ng/ml) was not associated with an significant increased risk of COVID-19 infection (OR for < 20 ng/ml: 1.61, 95% CI: 0.92–2.80, I2 = 92%) or in-hospital death (OR for < 20 ng/ml: 2.18, 95% CI: 0.91–5.26, I2 = 72%; OR for < 30 ng/ml: 3.07, 95% CI: 0.64–14.78, I2 = 66%) (Fig. 2B&D). When vitamin D level was analyzed as a continuous variable, each 10 ng/ml increase in vitamin D level was not associated with a significant decreased risk of COVID-19 inflection (OR: 0.92, 95% CI: 0.79–1.08, I2 = 98%) or death (OR: 0.65, 95% CI: 0.40–1.06, I2 = 79%) (Fig. 2A&C).

### Effect of vitamin D supplements on ICU admission or death

**Fig. 3:**
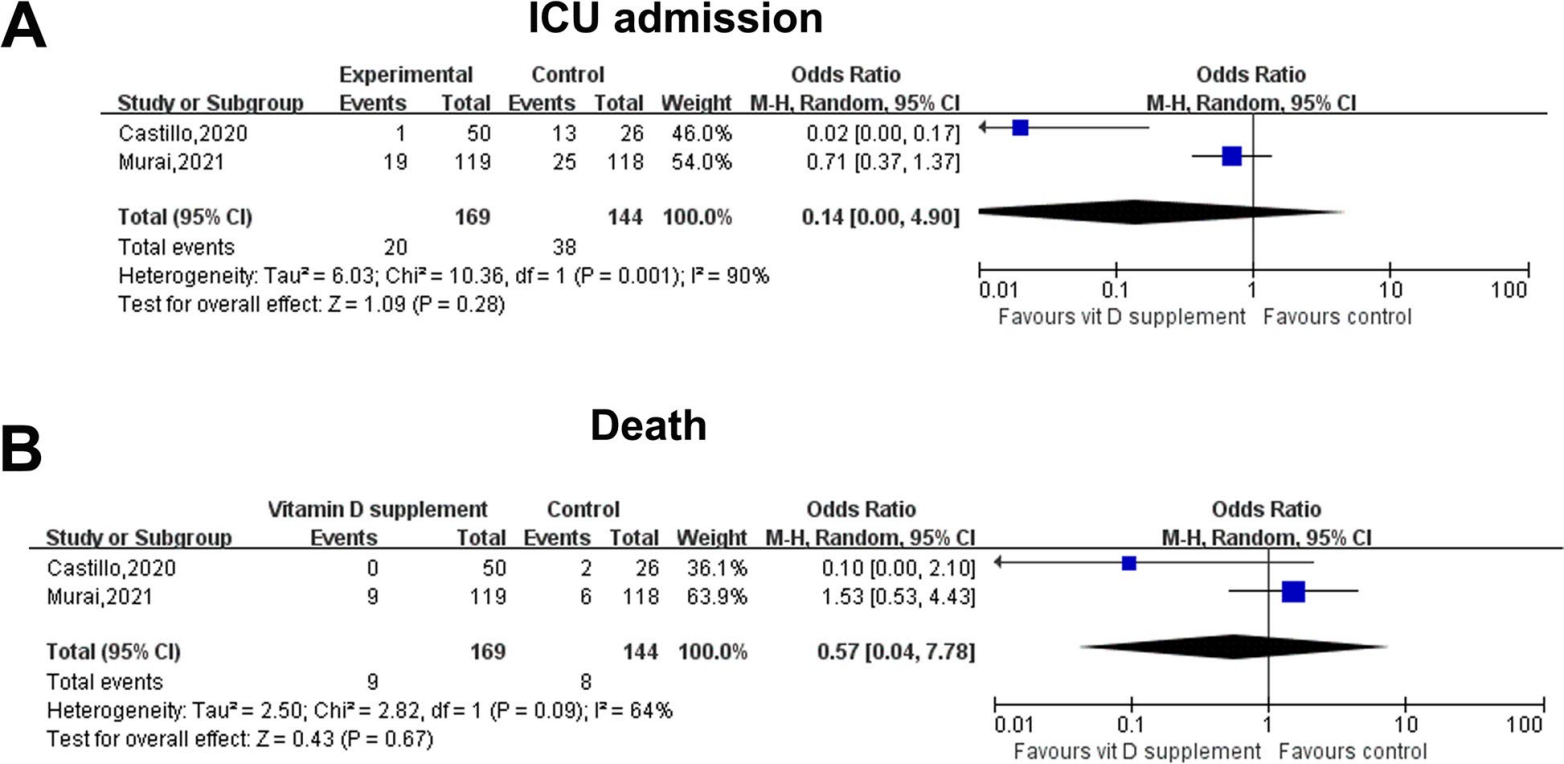
Forest plot showing the effect of vitamin D supplements on ICU admission and death in hospitalized patients with COVID-19. **A**: ICU admission; **B**: Death. Abbreviations: COVID-19, coronavirus disease 2019; OR, odds ratio; CI, confidence interval; IV, inverse variance; SE, standard error

Two [16, 20] RCTs including 233 hospitalized patients with COVID-19 (vitamin D supplement, *n* = 169; control *n* = 164) assessed the effect of vitamin D supplements on clinical outcomes in patients with COVID-19. Vitamin D supplements did not significantly decrease death (OR: 0.57, 95% CI: 0.04–7.78, I2 = 64%) or ICU admission (OR: 0.14, 95% CI: 0.00–4.90, I2 = 90%) in hospitalized patients with COVID-19 (Fig. 3).

### Publication bias

Publication bias was not assessed because of the limited studies (*n* < 10) according to the guidelines [25].

### Grade

The overall evidence for the RCTs and observational studies was qualified using GRADE. Very little confidence showed that low vitamin D supplementation contributed to an increased risk of COVID-19 or COVID-19-related death. Furthermore, only low-quality evidence supports the benefits of vitamin D supplementation on death or ICU admission in patients with COVID-19. The GRADE tables are described in detail in Supplemental Tables S6 and S7.

## Discussion

The present study showed that: (i) vitamin D deficiency (< 20 ng/ml) or insufficiency (< 30 ng/ml) was not associated with a significantly increased risk of COVID-19 infection or in-hospital death (*P* = 0.56). (ii) A 10 ng/ml increase in serum vitamin D was not significantly linked to an increased risk of COVID-19 infection or in-hospital death. (iii) Vitamin D supplements did not improve clinical outcomes in patients with COVID-19. Overall, our study suggested no significant association between vitamin D level, COVID-19 infection, and outcomes and no benefit of vitamin D supplementation in hospitalized patients with COVID-19.

### Comparisons with previous studies and further research

In contrast, several meta-analysis studies found a positive correlation between low serum vitamin D levels and worse clinical outcomes [26, 27]. However, they included case-control studies or reported unadjusted estimate effects, which might cause greater bias. For example, findings from the UK Biobank found a positive association between low vitamin D and COVID-19 infection, but the association was not significant after adjusting for confounders [6]. The present study included only cohort studies and multivariate-adjusted studies, which should reduce the potential bias. Evidence from a few Mendelian randomization studies also showed that vitamin D status did not causally affect susceptibility to and the severity of COVID-19 infection [28, 29]. Overall, these results strongly suggested no association between serum vitamin D and COVID-19.

Our results also support the recommendation of joint guidance of insufficient evidence of vitamin D for the treatment of COVID-19 [30]. Although a small random controlled trial showed that oral vitamin D supplementation helped achieve SARS-CoV-2 RNA negativity in greater proportion and decreased inflammatory markers [21], we did not find a significant benefit of vitamin D supplements by combining available evidence from RCTs on clinical outcomes in patients with COVID-19, which was consistent with two quasi-experimental studies [31, 32]. The overall quality of evidence for death or ICU admission was low, suggesting no recombination of vitamin D supplementation for hospitalized patients with COVID-19. However, all of the trials included a low or unclear percentage of patients with 25-hydroxyvitamin D deficiency. The benefits of vitamin D supplementation in patients with vitamin D deficiency should be further studied. Further studies with higher quality should also determine whether preventive or early vitamin D3 supplementation would be useful. (NCT04535791; NCT04482673; NCT04407286).

### Strengths and limitations

The strength of the present study lies in the study design, which included only cohort studies and RCTs. This study is the first meta-analysis to use the GRADE system to evaluate the quality of the evidence. The present study also has several limitations. Our meta-analysis had high heterogeneity, which might be derived from the study design and variability in baseline characteristics. For example, several studies have shown that the death rate for black patients with COVID-19 was higher than the rate for white patients with COVID-19 [33, 34]. Second, many factors modulate vitamin D status, including genetic polymorphisms, age, health, sun exposure behavior, and season [11]. Although we included only studies that performed multivariable analysis, some potential risk factors were not fully adjusted, which affected our results. Therefore, further research should adjust for additional confounding factors to verify the results.

## Conclusion

Based on current evidence, vitamin D deficiency or insufficiency was not significantly linked to susceptibility to COVID-19 infection or its associated death. Vitamin D supplements did not significantly improve clinical outcomes in patients with COVID-19, and the overall GRADE evidence quality was low, which suggested that vitamin D supplementation was not recommended for patients with COVID-19.

## Supplementary Information

**Additional file 1: Table S1. Table S2.** Detailed description of the search strategy. **Supplemental Table S4.** Quality assessment of the included observational studies. **Supplemental Table S5.** Quality assessment of the included RCT trials. **Table S6.** GRADE table observational studies. **Table S7.** GRADE table for RCTs.

## Data Availability

The datasets used and analyzed during the current study are available from the corresponding author on reasonable request.

## Abbreviations

OR: Odd ratio
COVID-19: Corona Virus Disease 2019
RAAS: Renin-angiotensin-aldosterone system
CIs: Confidence intervals
NOS: Newcastle-Ottawa quality assessment scale
