# Supplementary material for: Low vitamin D levels do not aggravate COVID-19 risk or death, and vitamin D supplementation does not improve outcomes in hospitalized patients with COVID-19: a meta-analysis and GRADE assessment of cohort studies and RCTs
Source: Nutr J. 2021 Oct 31;20:89. doi: 10.1186/s12937-021-00744-y (PMC8557713; doi:10.1186/s12937-021-00744-y)
Supplement: Supplementary file 1 — Additional file 1: Table S1. Table S2. Detailed description of the search strategy. Supplemental Table S4. Quality assessment of the included observational studies. Supplemental Table S5. Quality assessment of the included RCT trials. Table S6. GRADE table observational studies. Table S7. GRADE table for RCTs. [file 12937_2021_744_MOESM1_ESM.docx]

**Low vitamin D levels do not aggravate COVID‑19 risk or death, and vitamin D supplementation does not improve outcomes in hospitalized patients with COVID-19: A meta-analysis and GRADE assessment of cohort studies and RCTs**

| **Section/topic** | | **#** | | **Checklist item** | | **Reported on page #** | |
| --- | --- | --- | --- | --- | --- | --- | --- |
| **TITLE** | | | | | |  | |
| Title | | 1 | | Identify the report as a systematic review, meta-analysis, or both. | | 1 | |
| **ABSTRACT** | | | | | |  | |
| Structured summary | | 2 | | Provide a structured summary including, as applicable: background; objectives; data sources; study eligibility criteria, participants, and interventions; study appraisal and synthesis methods; results; limitations; conclusions and implications of key findings; systematic review registration number. | | 3 | |
| **INTRODUCTION** | | | | | |  | |
| Rationale | | 3 | | Describe the rationale for the review in the context of what is already known. | | 5 | |
| Objectives | | 4 | | Provide an explicit statement of questions being addressed with reference to participants, interventions, comparisons, outcomes, and study design (PICOS). | | 5 | |
| **METHODS** | | | | | |  | |
| Protocol and registration | | 5 | | Indicate if a review protocol exists, if and where it can be accessed (e.g., Web address), and, if available, provide registration information including registration number. | | Not applicable | |
| Eligibility criteria | | 6 | | Specify study characteristics (e.g., PICOS, length of follow-up) and report characteristics (e.g., years considered, language, publication status) used as criteria for eligibility, giving rationale. | | 5-6 | |
| Information sources | | 7 | | Describe all information sources (e.g., databases with dates of coverage, contact with study authors to identify additional studies) in the search and date last searched. | | 5 | |
| Search | | 8 | | Present full electronic search strategy for at least one database, including any limits used, such that it could be repeated. | | 5 | |
| Study selection | | 9 | | State the process for selecting studies (i.e., screening, eligibility, included in systematic review, and, if applicable, included in the meta-analysis). | | 5-6 | |
| Data collection process | | 10 | | Describe method of data extraction from reports (e.g., piloted forms, independently, in duplicate) and any processes for obtaining and confirming data from investigators. | | 5-6 | |
| Data items | | 11 | | List and define all variables for which data were sought (e.g., PICOS, funding sources) and any assumptions and simplifications made. | | 5 | |
| Risk of bias in individual studies | | 12 | | Describe methods used for assessing risk of bias of individual studies (including specification of whether this was done at the study or outcome level), and how this information is to be used in any data synthesis. | | 6 | |
| Summary measures | | 13 | | State the principal summary measures (e.g., risk ratio, difference in means). | | 6 | |
| Synthesis of results | | 14 | | Describe the methods of handling data and combining results of studies, if done, including measures of consistency (e.g., I^2^) for each meta-analysis. | | 6 | |
| Risk of bias across studies | | 15 | | Specify any assessment of risk of bias that may affect the cumulative evidence (e.g., publication bias, selective reporting within studies). | | 6 | |
| Additional analyses | | 16 | | Describe methods of additional analyses (e.g., sensitivity or subgroup analyses, meta-regression), if done, indicating which were pre-specified. | | Not performed | |
| **RESULTS** | | | | |  | |  |
| Study selection | 17 | | Give numbers of studies screened, assessed for eligibility, and included in the review, with reasons for exclusions at each stage, ideally with a flow diagram. | | 6 | |  |
| Study characteristics | 18 | | For each study, present characteristics for which data were extracted (e.g., study size, PICOS, follow-up period) and provide the citations. | | 6-7 | |  |
| Risk of bias within studies | 19 | | Present data on risk of bias of each study and, if available, any outcome level assessment (see item 12). | | 7 | |  |
| Results of individual studies | 20 | | For all outcomes considered (benefits or harms), present, for each study: (a) simple summary data for each intervention group (b) effect estimates and confidence intervals, ideally with a forest plot. | | 7 | |  |
| Synthesis of results | 21 | | Present results of each meta-analysis done, including confidence intervals and measures of consistency. | | 7 | |  |
| Risk of bias across studies | 22 | | Present results of any assessment of risk of bias across studies (see Item 15). | | 7 | |  |
| Additional analysis | 23 | | Give results of additional analyses, if done (e.g., sensitivity or subgroup analyses, meta-regression [see Item 16]). | | 7 | |  |
| **DISCUSSION** | | | | |  | |  |
| Summary of evidence | 24 | | Summarize the main findings including the strength of evidence for each main outcome; consider their relevance to key groups (e.g., healthcare providers, users, and policy makers). | | 8 | |  |
| Limitations | 25 | | Discuss limitations at study and outcome level (e.g., risk of bias), and at review-level (e.g., incomplete retrieval of identified research, reporting bias). | | 9 | |  |
| Conclusions | 26 | | Provide a general interpretation of the results in the context of other evidence, and implications for future research. | | 10 | |  |
| **FUNDING** | | | | |  | |  |
| Funding | 27 | | Describe sources of funding for the systematic review and other support (e.g., supply of data); role of funders for the systematic review. | | 10 | |  |

**Table S2:** Detailed description of the search strategy

**PubMed**

| Search | Query |
| --- | --- |
| #1 | SARS-COV2 |
| #2 | COVID-19 |
| #3 | 2019-nCoV |
| #4 | 2019-novel coronavirus |
| #5 | ergocalciferol |
| #6 | cholecalciferol |
| #7 | vitamin D |
| #8 | 25(OH)D |
| #9 | vitamin D3 |
| #11 | 25-hydroxycholecalciferol |
| #12 | death |
| #13 | mortality |
| #14 | Intensive care unit |
| #15 | Severe |
| #16 | severity |
| #17 | #1 OR #2 OR #3 OR #4 |
| #18 | #5 OR #6 OR #7 OR #8 OR #9 OR #10 OR #11 |
| #19 | #12 OR #13 OR #13 OR #14 OR #15 OR #16 |
| #20 | #17 AND #18 AND #19 |

**Embase**

| Search | Query |
| --- | --- |
| #1 | SARS-COV2 |
| #2 | coronavirus disease 2019 |
| #3 | ergocalciferol |
| #4 | cholecalciferol |
| #5 | vitamin D |
| #6 | 25(OH)D |
| #7 | vitamin D3 |
| #8 | calcifediol |
| #9 | death |
| #10 | mortality |
| #11 | Intensive care unit |
| #12 | Severe |
| #13 | severity |
| #14 | #1 OR #2 |
| #15 | #3 OR #4 OR #5 OR #8 OR |
| #16 | #9 OR #10 OR #11 OR #12 OR #13 |
| #17 | #14 AND #15 AND #16 |
|  |  |

**medRxiv**

| Search | Query |
| --- | --- |
| #1 | SARS-COV2 |
| #2 | COVID-19 |
| #3 | 2019-nCoV |
| #4 | 2019-novel coronavirus |
| #5 | ergocalciferol |
| #6 | cholecalciferol |
| #7 | vitamin D |
| #8 | 25(OH)D |
| #9 | vitamin D3 |
| #11 | 25-hydroxycholecalciferol |
| #12 | death |
| #13 | mortality |
| #14 | Intensive care unit |
| #15 | Severe |
| #16 | severity |
| #17 | #1 OR #2 OR #3 OR #4 |
| #18 | #5 OR #6 OR #7 OR #8 OR #9 OR #10 OR #11 |
| #19 | #12 OR #13 OR #13 OR #14 OR #15 OR #16 |
| #20 | #17 AND #18 AND #19 |

**Supplemental Table S4. Quality assessment of the included observational studies**

| **Author**  **(Publication Year)** | **Newcastle-Ottawa Scale** | | | | | | | | | |
| --- | --- | --- | --- | --- | --- | --- | --- | --- | --- | --- |
|  | **Selection** | | | **Comparability** | | | **Outcome** | | | **Total** |
|  | **a** | **b** | **c** | **d** | **e** | **f** | **g** | **h** | **i** |  |
| Hastie, 2020, UK | 1 | 1 | 1 | 1 | 1 | 1 | 1 | 1 | 1 | 9 |
| Radujkovic,2020, Germany | 1 | 1 | 1 | 1 | 1 | 1 | 1 | 1 | 0 | 8 |
| De Smet,2021, Germany | 1 | 1 | 1 | 1 | 1 | 1 | 1 | 1 | 1 | 9 |
| AlSafar, 2021, Australia | 1 | 1 | 1 | 1 | 1 | 1 | 1 | 1 | 1 | 9 |
| Charoenngam,2021, USA | 1 | 1 | 1 | 1 | 1 | 1 | 1 | 1 | 0 | 8 |
| Carpagnano,2020, Italy | 1 | 1 | 1 | 1 | 0 | 1 | 1 | 1 | 0 | 7 |
| Katz D,2020, USA | 1 | 1 | 1 | 1 | 1 | 1 | 1 | 1 | 1 | 9 |
| Kaufman, 2020, USA | 1 | 1 | 1 | 1 | 1 | 1 | 1 | 1 | 1 | 9 |
| Angelidi,2020, USA | 0 | 1 | 1 | 1 | 1 | 1 | 1 | 1 | 1 | 8 |

1. Representativeness of the exposed cohort.
2. Selection of the non-exposed cohort.
3. Ascertainment of exposure.
4. Demonstration that outcome of interest was not present at start of study.
5. Comparability of cohorts on the basis of the design or analysis (adjusted for age).
6. Comparability of cohorts on the basis of the design or analysis (adjusted for any other factor).
7. Assessment of outcome.
8. Was follow-up long enough for outcomes to occur. (>5 years).
9. Adequacy of follow-up of cohorts.

**
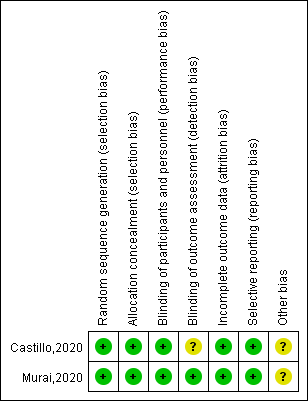
**

**Supplemental Table S5. Quality assessment of the included RCT trials**

**Table S6: GRADE table observational studies.**

**Author(s)**:

| **№ of studies** | **Certainty assessment** | | | | | | **Effect** | | | **Certainty** | **Importance** |
| --- | --- | --- | --- | --- | --- | --- | --- | --- | --- | --- | --- |
|  | **Study design** | **Risk of bias** | **Inconsistency** | **Indirectness** | **Imprecision** | **Other considerations** | **№ of events** | **№ of individuals** | **Rate (95% CI)** |  |  |
| Risk of COVID-19 | | | | | | | | | | | |
|  | observational studies | not serious | very serious ^a^ | serious ^b^ | not serious ^c^ | none |  |  |  | ⨁◯◯◯ VERY LOW | IMPORTANT |
| Death | | | | | | | | | | | |
|  | observational studies | not serious | very serious ^a^ | serious ^b^ | not serious ^c^ | none |  |  |  | ⨁◯◯◯ VERY LOW | CRITICAL |

#### Explanations

a. large heterogeneity

b. Vitamin D level was not tested at enrollment

c. large heterogeneity

**Table S7: GRADE table for RCTs**

| **Certainty assessment** | | | | | | | **№ of patients** | | **Effect** | | **Certainty** | **Importance** |
| --- | --- | --- | --- | --- | --- | --- | --- | --- | --- | --- | --- | --- |
| **№ of studies** | **Study design** | **Risk of bias** | **Inconsistency** | **Indirectness** | **Imprecision** | **Other considerations** | **vitamin D supplement** | **control** | **Relative (95% CI)** | **Absolute (95% CI)** |  |  |
| **Death** | | | | | | | | | | | | |
| 0 | Randomized trials | not serious | serious ^a^ | not serious | serious ^b^ | publication bias strongly suspected all plausible residual confounding would reduce the demonstrated effect | 9/169 (5.3%) | 8/144 (5.6%) | **OR 0.57** (0.04 to 7.78) | **23 fewer per 1,000** (from 53 fewer to 258 more) | ⨁⨁◯◯ LOW | CRITICAL |
| **ICU admission** | | | | | | | | | | | | |
| 0 | Randomized trials | not serious | serious ^c^ | not serious | very serious ^d^ | publication bias strongly suspected very strong association | 20/169 (11.8%) | 38/144 (26.4%) | **OR 0.14** (0.00 to 4.19) | **216 fewer per 1,000** (from -- to 336 more) | ⨁⨁◯◯ LOW | IMPORTANT |

**CI:** Confidence interval; **OR:** Odds ratio

#### Explanations

a. large heterogeneity

b. low numbers of cases

c. large heterogeneity
